# Rapid Geographical Origin Identification and Quality Assessment of Angelicae Sinensis Radix by FT-NIR Spectroscopy

**DOI:** 10.1155/2021/8875876

**Published:** 2021-01-12

**Authors:** Zhen-yu Zhang, Ying-jun Wang, Hui Yan, Xiang-wei Chang, Gui-sheng Zhou, Lei Zhu, Pei Liu, Sheng Guo, Tina T. X. Dong, Jin-ao Duan

**Affiliations:** ^1^National and Local Collaborative Engineering Center of Chinese Medicinal Resources Industrialization and Formulae Innovative Medicine, and Jiangsu Collaborative Innovation Center of Chinese Medicinal Resources Industrialization, Nanjing University of Chinese Medicine, Nanjing 210023, China; ^2^School of Pharmacy, Anhui University of Chinese Medicine, Hefei 230012, China; ^3^Division of Life Science and Centre for Chinese Medicine, The Hong Kong University of Science and Technology, Hong Kong, China

## Abstract

Angelicae Sinensis Radix is a widely used traditional Chinese medicine and spice in China. The purpose of this study was to develop a methodology for geographical classification of Angelicae Sinensis Radix and determine the contents of ferulic acid and Z-ligustilide in the samples using near-infrared spectroscopy. A qualitative model was established to identify the geographical origin of Angelicae Sinensis Radix using Fourier transform near-infrared (FT-NIR) spectroscopy. Support vector machine (SVM) algorithms were used for the establishment of a qualitative model. The optimum SVM model had a recognition rate of 100% for the calibration set and 83.72% for the prediction set. In addition, a quantitative model was established to predict the content of ferulic acid and Z-ligustilide using FT-NIR. Partial least squares regression (PLSR) algorithms were used for the establishment of a quantitative model. Synergy interval-PLS (Si-PLS) was used to screen the characteristic spectral interval to obtain the best PLSR model. The coefficient of determination for calibration (R2C) for the best PLSR models established with the optimal spectral preprocessing method and selected important spectral regions for the quantitative determination of ferulic acid and Z-ligustilide was 0.9659 and 0.9611, respectively, while the coefficient of determination for prediction (R2P) was 0.9118 and 0.9206, respectively. The values of the ratio of prediction to deviation (RPD) of the two final optimized PLSR models were greater than 2. The results suggested that NIR spectroscopy combined with SVM and PLSR algorithms could be exploited in the discrimination of Angelicae Sinensis Radix from different geographical locations for quality assurance and monitoring. This study might serve as a reference for quality evaluation of agricultural, pharmaceutical, and food products.

## 1. Introduction

*Angelica sinensis* (Oliv.) Diels is a perennial plant that is widely found in China, Korea, and Japan. The dried root of *A. sinensis* (Oliv.) Diels (Angelicae Sinensis Radix (ASR), Danggui in Chinese) has a long history of use in China. It was used not only as a spice or therapeutic food (cooked with meat and taken as soup) but also as traditional Chinese medicine (TCM) for replenishing and invigorating blood, relieving pain, and moistening the intestines [1]. ASR is often cooked with lamb, for instance, *Danggui Shengjiang Yangrou Tang*. This recipe was first recorded in *Jin Gui Yao Lue* written by *Zhang Zhongjing* [2]. ASR is currently available as a food/dietary supplement in North America and Europe and is known by the standardized generic name Dong quai [3]. Recently, pharmacological studies have revealed that ASR has several bioactivities, including anti-arrhythmic and anti-atherosclerotic, as well as preventing myocardial infarction events, and protecting the heart [4–7]. Additionally, ASR is predominantly renowned for its outstanding effects in the treatment of various gynecological conditions that are generally difficult to treat with conventional therapies; hence, it is also known as “female ginseng” [8].

Modern research has shown that the geographical origin of agricultural products has a direct impact on the quality and safety of agricultural products [9, 10]. Reliable information regarding geographical origin is considered the most critical factor for consumers when purchasing agricultural products [11]. Hence, traceability of the geographical origin of food products is one of the emerging and critical issues in the agricultural sector. ASR is mainly distributed in Gansu, Qinghai, Yunnan, and other provinces in China. Its quality is quite different across different geographical origins. For instance, ASR produced in Minxian County, Gansu Province, China, has the best quality and clinical effects as “*daodi* herb,” according to traditional experience [12]. In the international and domestic markets, “*mingui*” was sold at higher prices than the ASR from other geographical origins. Currently, “*mingui*” is facing an adulteration problem similar to other premium-priced foodstuffs due to its growing demand [13]. Deceitful activities, such as selling ASR from other geographical origins with lower quality and price as *mingui*, are conducted by dishonest businesspersons to increase their profit margin. Therefore, to control and secure the quality of *danggui*, as well as trace its geographical origin, the analysis of ASR has become almost indispensable and mandatory.

It is difficult to distinguish food products or medicinal materials from different geographical origins with the naked eye. To date, various methods have been applied in the traceability of food products, including near-infrared spectroscopy (NIR), isotope ratio mass spectrometry (IRMS), inductively coupled plasma mass spectrometry (ICP-MS), high-performance liquid chromatography (HPLC), liquid chromatography coupled with tandem mass spectrometry (LC-MS/MS), among other analytical methods [14–17]. Fourier transform near-infrared spectroscopy (FT-NIR), a novel quality control technology, has been increasingly introduced as a fast and routinely applied method in qualitative and quantitative analysis of plant raw materials, because it affords simple sample preparation and rapid simultaneous analysis of several analytes in a large number of samples, as well as geographical origin classification [18, 19]. At present, near-infrared (NIR) spectroscopy technology has been widely used to trace geographical origins of seafood, wine, TCM, etc. [20–22]. It has shown outstanding potentials in agriculture, food, pharmacy, the environment, and many other fields.

Ferulic acid and Z-ligustilide have been widely considered as the main active components in ASR with various pharmacological effects, including antithrombotic effects, antidepressant-like effects, and anti-atherosclerosis, neuroprotective, anti-cancer, anti-inflammatory, and vasodilator effects [23–27]. Ferulic acid and Z-ligustilide have also been commonly used as index ingredients to evaluate the quality of ASR [28]. Although conventional detection methods such as chromatography are sufficiently sensitive, they are often time-consuming and labor-intensive and require expensive equipment, involving elaborate organic solvent extraction. Therefore, a rapid, cheap, environmentally friendly, and comprehensive method is needed to quantitatively detect the content of ferulic acid and Z-ligustilide in ASR to assess the quality of ASR.

At present, there have been reports on the application of NIR in the identification and content prediction of ASR. Li et al. [29] established a qualitative model of ASR by random forest (RF) to identify the geographical origins of ASR and constructed a quantitative model of the ethanol extract and ferulic acid of ASR using the genetic algorithm optimization combined with multiple linear regression (GA-MLR) method. However, the quantitative model constructed by Li and colleagues was not reliable and needed to be improved. Besides, they did not construct the quantitative model of Z-ligustilide. Z-ligustilide has a wide range of pharmacological properties, including anticancer, anti-inflammatory, antioxidant, neuroprotective activities, etc. [30]. Z-ligustilide is also the main indicator of quality control of TCM compounds containing ASR and essential oil of ASR [12]. In addition to RF and GA-MLR, many other multivariate statistical techniques can complement NIR spectrum analysis. For example, a support vector machine (SVM) is a commonly used algorithm for building qualitative models. It is a useful tool for SVM to analyze the data that are not regularly distributed or have an unknown distribution [31]. SVM is also a useful classification technique when few training data are available [32]. Geographical origin traceability studies on teas and *Panax notoginseng* showed that an SVM classifier was better than other classifiers [33, 34]. A study by Xu et al. [35] showed that NIR spectroscopy coupled with the SVM model had a good effect on the identification of the origin of TCM. Partial least squares regression (PLSR) is a classical and widely used linear method for modeling spectral data. Studies have shown that the prediction accuracies of PLS-derived models tend to be higher than that of multiple regression- (MR-) derived models [36, 37]. Our previous study found that the PLSR model had excellent predictive and extrapolation abilities [19, 38].

Therefore, the present study aimed to explore the feasibility of the FT-NIR spectroscopy technique with SVM in discrimination of the geographical origin of ASR. Additionally, the capability of determining the content of ferulic acid and Z-ligustilide in ASR using NIR spectroscopy coupled with PLSR calibration was also investigated. Eight preprocessing methods were compared to select the favorable one, and then Synergy interval-PLS (Si-PLS) was used to screen the characteristic spectral interval to obtain the best PLSR model. This study might serve as a reference for the rapid geographical origin, identification, and quality control of ASR.

## 2. Materials and Methods

### 2.1. Samples and Reagents

Ninety-nine batches of ASR were purchased from formal Chinese medicinal material markets or medicinal material planting cooperatives in Qinghai, Yunnan, and Gansu provinces of China. Twenty batches of ASR were from Qinghai, twenty-five were from Yunnan, and fifty-four were from Gansu. Their botanical origins were authenticated as *Angelica sinensis* (Oliv.) Diels by Professor Hui Yan. Voucher specimens were deposited at the Herbarium of Jiangsu Collaborative Innovation Center of Chinese Medicinal Resources Industrialization. Dried samples were pulverized to homogeneous powders (50 mesh) for HPLC and NIR analyses.

Standards of ferulic acid and Z-ligustilide (Batch No. 20180302-29 and 18113001, respectively; purity >98% for both) were provided by the National Institutes for Food and Drug Control (Beijing, China) and Nanjing Jin Yibai Biological Technology Co. Ltd. (Nanjing, China), respectively. Methanol and acetonitrile were HPLC grade and purchased from Merck (Darmstadt, Germany). Water was purified using an ultrapure water instrument. All other solvents and reagents were of analytical grade unless otherwise noted.

### 2.2. Acquisition of Near Infrared Spectra

FT-NIR spectra were collected with a diffuse reflection module using an Antaris™ II FT-NIR spectrophotometer (Thermo Fisher Scientific Co., China) equipped with a rotating sample-cup spinner, an extended InGaAs detector, and a tungsten halogen lamp as the light source. Result Software (Antaris™ II System, Thermo Fisher Scientific Co., China) was used in FT-NIR data acquisition. The spectral data were recorded as the average of 32 scans in the spectral range of 10,000-4,000 cm^−1^ and the spectra were collected at a spectral resolution of 8 cm^−1^, with air absorbance as the reference standard at room temperature (25°C). To improve the accuracy of the collected spectral data, the spectra of each sample were recorded in triplicate and the average spectrum was used for subsequent analysis.

### 2.3. Determination of Reference Values by HPLC

About 0.2 g of the sample powder was weighed and extracted with 20 mL 70% methanol in a 50 mL glass-stoppered conical ﬂask. All mixtures were precisely weighed and then extracted by a heating reflux apparatus for 30 min. After the solution was cooled, the mixture was weighed again, and the lost weight was made up of 70% methanol. After centrifugation at 13,000 r/min for 10 min, the supernatants were stored at 4°C on a sample plate and filtered through 0.22 *µ*m cellulose membrane filters before HPLC analysis. The standard substances of ferulic acid and Z-ligustilide were precisely weighed and acetonitrile was added to prepare the standard stock solution with a concentration of 1.3543 and 2.2011 mg/mL, respectively. Certain amounts of standard stock solutions were mixed and diluted with acetonitrile to aﬀord a series of solutions at appropriate concentrations, which were used to construct calibration curves. Prior to use, the mixed standard solution was filtered using 0.22 *µ*m cellulose membrane filters before analysis and stored at 4°C.

The validation of the analytical method was performed by evaluating linearity, precision, repeatability, stability, and recovery. The mixed standard solution was diluted to a series of solutions with at least six appropriate concentrations in duplicate to make calibration curves, and then a linear regression was constructed by plotting the peak areas versus the corresponding concentration of each analyte. Precision was evaluated by analyzing the mixed standard solution six times. To evaluate repeatability, six sample solutions were independently prepared from the same batch of ASR samples and analyzed in parallel. To evaluate the sample stability, the sample solution aforementioned was stored at 25°C and, respectively, determined at various periods (0, 2, 4, 8, 12, and 24 h). All these variations were expressed as relative standard deviation (RSD). Recovery was performed by spiking known quantities of ferulic acid and Z-ligustilide with high (150%), middle (100%), and low (50%) levels to a certain amount of ASR samples that had been analyzed in the repeatability test. Then, the spiked samples were extracted, processed, and quantified based on previously described methods. Triplicate experiments were performed at each level.

HPLC analysis was conducted on a Waters Alliance E2695 HPLC system (Waters, Milford, MA, USA) equipped with a Waters 2998 PDA detector. Every sample was separated on a Waters Symmetry C18 column (4.6 mm × 250 mm, 5 *μ*m) with column temperature at 30°C. The mobile phase system consisting of acetonitrile (A) and 0.5% acetic acid aqueous solution (B) was applied with a gradient elution of 5-45% A at 0–25 min, 45–55% A at 25–35 min, 55% A at 35–45 min, 55–95% A at 45–55 min, and 95% A at 55–60 min. The detection wavelength was 320 nm. The injection volume was 10 *μ*L and the flow rate was set at 1.0 mL/min.

### 2.4. Spectral Processing

Ninety-nine batches of ASR samples from three different geographical origins were randomly categorized into the calibration set and prediction set in a 2 : 1 ratio using the SPXY algorithm. Sixty-six samples were used for the calibration set to develop the models and the remaining 33 samples comprised the prediction set for testing the performance of the established models. The raw NIR spectra contain lots of chemical information of the samples; however, background information and systematic noise unrelated to the properties of the test sample exist at the same time due to the inﬂuences of light scattering, path length difference, sample particle size, and other factors [39, 40]. Therefore, it was necessary to use spectral pretreatment methods to remove the extraneous variables that did not represent the actual target chemical compositions. First, the raw FT-NIR spectra were preprocessed using OMNIC software for averaging spectroscopy, normalization of longitudinal coordinates. Then, eight spectral preprocessing methods, namely, multiplicative scatter correction (MSC), standard normal variate transformation (SNV), first derivative (Savitzky–Golay algorithm with 11 points of smoothing, 1D), second derivative (Savitzky–Golay algorithm with 11 points of smoothing, 2D), MSC + 1D, MSC + 2D, SNV + 1D, and SNV + 2D, were compared to optimize FT-NIR data. MSC can effectively eliminate physical effects such as particle size and surface blaze, which do not carry any chemical or physical information. This method can correct differences in the baseline and the trend and has the advantage that the transformed spectra are similar to the original spectra [41]. SNV is also a preprocessing approach that can remove multiplicative interferences of the scatter and particle size and is commonly used in solid diffuse reflectance and slurry transmittance spectra [42]. NIR diffuse reflectance spectra transposed by SNV have no multicollinearity and are not confused by the shape complexity encountered with the use of derivative spectroscopy [43]. Derivatives (1D and 2D) can remove the baseline drifts, separate broad and overlapping NIR bands, eﬀects of noise, improve the spectral difference, and preserve the relative band-intensity information without significantly increasing the spectral noise [44, 45].

### 2.5. Support Vector Machine (SVM)

SVM is a non-linear supervised classification model that can be used for pattern classification and nonlinear regression [46]. The SVM-based classification model is characterized by two parameters, *C* (the penalty parameter) and *g* (the kernel width parameter), which should be carefully selected to achieve good performance [18]. In this study, the combination of parameters *C* and *g* of the SVM model was calculated by the approach of the grid search (GS) method with 10-fold cross-validation.

### 2.6. Partial Least Squares Regression (PLSR)

PLSR is a powerful statistical technique in constructing calibration models with infrared (IR) spectral data that can be used to explore the relationship between independent variables and dependent variables based on the reduction of the dimensionality of the data set [19]. The following parameters were calculated to assess the success of data preprocessing and model performance: coefficient of determination for calibration (*R*^2^_C_) and prediction (*R*^2^_P_), root mean square error of estimation (RMSEE), root mean square error of cross-validation (RMSECV), root mean square error of prediction (RMSEP), and the ratio of prediction to deviation (RPD). The coefficient of determination of *R*^2^_C_ and *R*^2^_P_ close to 1 indicates a good relationship between the predicted and measured values in the calibration and prediction sets [19]. RMSECV, RMSEE, and RMSEP are usually used to, respectively, evaluate the error of calibration and prediction sets. RMSECV based on a 7-fold cross-validation procedure was used to evaluate the modeling capacity of the PLSR model using the calibration set. RPD reﬂects the overall predictive ability of a PLSR model. In practical applications, performance is considered good when the RPD value is greater than 2 [47].

### 2.7. Software

The raw FT-NIR spectra were normalized by OMNIC 8.0 (Thermo Fisher Scientific Inc., Waltham, MA, USA). The spectral pretreatment and qualitative discrimination models of SVM were conducted using MATLAB software (version R2017a, MathWorks, Natick, USA) in Windows 8.1. SIMCA-P + program (version 14.1, Umetrics, Sweden) was used to establish the PLSR quantitative model. ORIGIN 8.0 (Northampton, MA, USA) was used for drawing.

## 3. Results and Discussions

### 3.1. NIR Spectral Features

**Figure 1:**
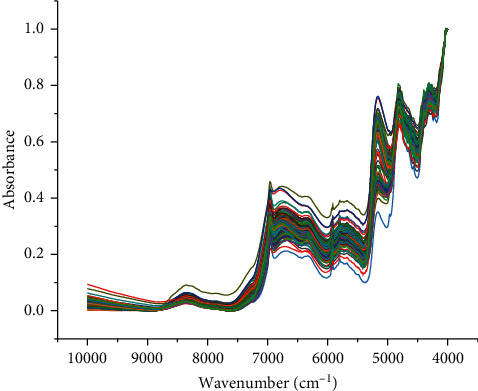
Raw NIR spectra of 99 ASR samples.

**Figure 2:**
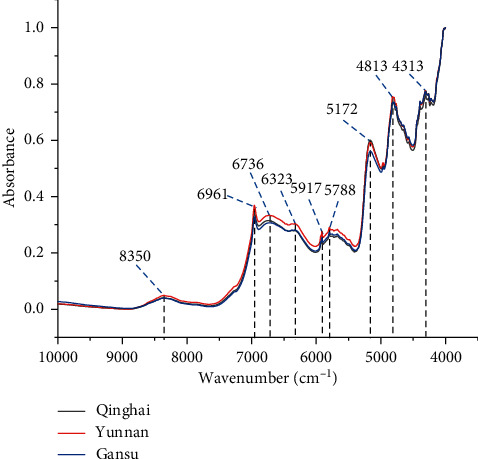
Average raw NIR spectra of ASR samples from three different geographical origins.

**Figure 3:**
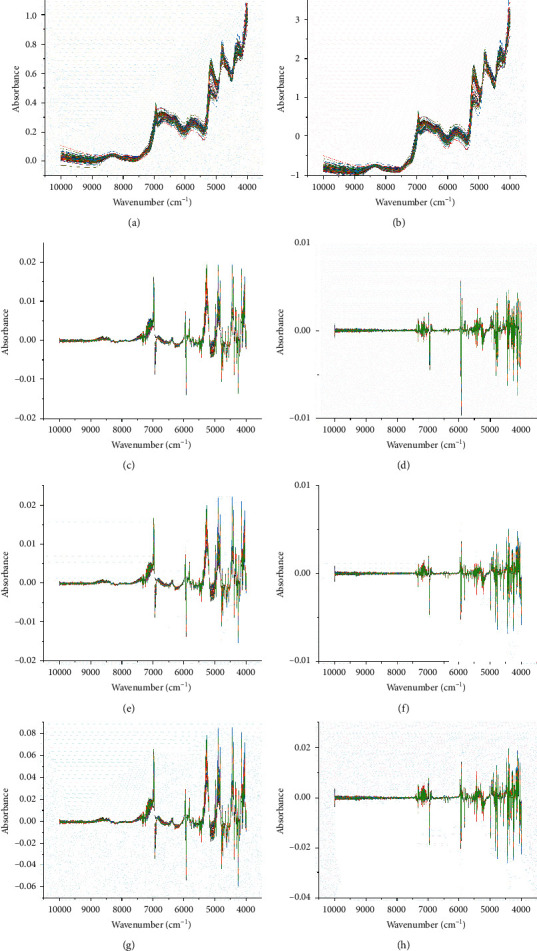
Spectra of different spectral preprocessing methods. (a) Multiplicative scatter correction (MSC). (b) Standard normal variate transformation (SNV). (c) First derivative (Savitzky–Golay algorithm with 11 points of smoothing, 1D). (d) Second derivative (Savitzky–Golay algorithm with 11 points of smoothing, 2D). (e) MSC + 1D. (f) MSC + 2D. (g) SNV + 1D. (h) SNV + 2D.

The raw NIR spectra of 99 ASR samples are shown in Figure 1. The FT-NIR spectra reflect valuable chemical information of the major secondary metabolites of ASR [48, 49]. As shown in Figure 2, comparing the FT-NIR spectra of ASR from the three diﬀerent geographical origins, the mean spectra from Qinghai, Yunnan, and Gansu were overlapping, and the peak information such as the peak height and peak intensity was similar in the map. They showed multiple common absorption bonds, including those at 8350, 6961, 6736, 6323, 5917, 5788, 5712, 4813, and 4313 cm^−1^. The peak around 8350 cm^−1^ was induced by the second overtone of C-H stretching, and the bands in the region of 6000-7000 cm^−1^ were assigned to the first overtone of the O-H and N-H stretching [33]. The bands in the region of 5500-6000 cm^−1^ belong to the first overtone of C-H stretching vibrations [19]. Broad band at 5172 cm^−1^ was mainly induced by the combination of O-H and C-O stretching, and the peaks around 4813 cm^−1^ were attributed to the combination of O-H bending and C-O stretching.  Figure 3 shows the spectra pretreated with the eight reprocessing methods.

### 3.2. Support Vector Machine Model

The main idea of SVM is to map input vectors to high-dimensional space and construct a maximal classification hyperplane that allows linear separation in the higher dimensional feature space, when the linear boundary in the low dimension input space is not enough to separate the different groups [50, 51]. In SVM, transformation into higher dimensional space can be realized through a kernel function. There are five possible choices for the kernel function options, including linear, polynomial, radial basis function (RBF), sigmoid, and precomputed, and the selection of kernel functions is of great importance in the performance of SVM [31, 35]. Previous studies have shown that SVM based on RBF kernel has excellent classification performance; thus, RBF kernel was selected as the kernel function of SVM classification in our study [31, 52]. The parameters to be optimized in the RBF kernel include the penalty parameter C and the kernel function parameter *g*. The RBF kernel parameter *g* defines the width of the kernel, which reduces the computational complexity of the training procedure and gives a good performance under general smoothness assumptions [33]. The regularization parameter C controls the trade-off between minimizing the model complexity and minimizing the training error.

**Figure 4:**
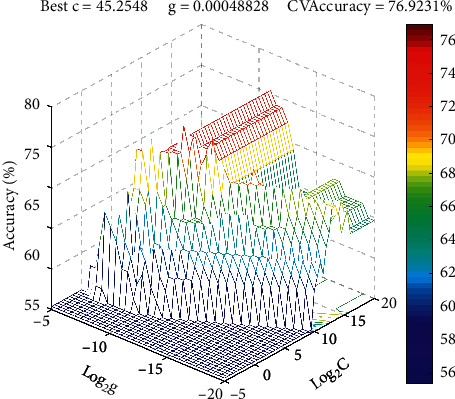
The optimization results for parameters C and g by GS methods with a 10-fold cross-validation.

The efficiency of SVM depends on the optimization of the regularization parameter C and RBF kernel parameter *g* [18]. In this study, the combination of parameters C and *g* of the SVM model was calculated using GS method approach with 10-fold cross-validation. When the highest classification accuracy was achieved, the best *C* and *g* were selected. The results of the optimization of the spectral pretreatment methods are shown in Table 1. The results show that the multiplicative scatter correction combined with the first derivative has the best performance for the SVM model. As shown in Figure 4, the 3D plot shows the different classification accuracy of the SVM model influenced by the values of Log2 C and Log2 g under 10-fold cross-validation, with the values of Log2 *C* ranging from −5 to 20, and those of Log2 *g* ranging from −20 to −5; the process of searching starts with a granularity of 0.5. The best parameter for cross verification was achieved when parameters *C* = 45.2548 and *g*= 0.00048828, with the highest cross validation (CV) accuracy value of 76.9231%. Subsequently, the calibration set was used for training of the SVM model and the prediction set was used to verify the accuracy of the established model using the optimal parameters *C* and *g*. The recognition rates for calibration and prediction sets were 100% and 83.72%, respectively. The results indicated that the performance of SVM model was able to identify the geographical origin of ASR.

### 3.3. Predicting the Content of Ferulic Acid and Z-Ligustilide Based on FT-NIR Using PLSR Algorithm

The results of the SVM classification model showed that NIR could distinguish the geographical origin of ASR, with a recognition rate of 83.72%. The information contained in the NIR spectra can be used not only to identify the geographical origins but also for rapid quantitative analysis of the components in ASR. To further explore the application of NIR in the rapid quantitative detection of ASR, the contents of ferulic acid and Z-ligustilide in ASR were estimated using NIR spectroscopy combined with the PLSR calibration model.

#### 3.3.1. High-Performance Liquid Chromatography Analysis

For quantitative considerations, the proposed HPLC method was validated by evaluating linearity, precision, repeatability, stability, and recovery. The results of method validation for HPLC analysis of ferulic acid and Z-ligustilide are shown in Table 2. The calibration curves of ferulic acid and Z-ligustilide exhibited good linear regression with correlation coefficient values (*r*^2^) of 0.9999, for both, and the concentration ranges between 0.5417–54.1728 *μ*g/mL and 3.7418-374.1836 *μ*g/mL, respectively. The RSD values for precision, repeatability, and storage stability were all not more than 3.0%. The recoveries were 99.10% and 97.00% for ferulic acid and Z-ligustilide, respectively, which showed that the method was accurate enough. Taken together, these results indicated that the developed HPLC method was sensitive, repeatable, and accurate for quantitative analysis.  Table 3 shows the results of HPLC content determination. The reference value of ferulic acid ranged from 0.4790 to 2.7307 mg/g and 0.4730 to 1.5626 mg/g in the calibration set and prediction set, respectively. The reference value of Z-ligustilide ranged from 2.2710 to 19.6627 mg/g and 3.4730 to 18.6352 mg/g in the calibration set and prediction set, respectively. The content of ferulic acid and Z-ligustilide had a wide range of distribution and good representation, which can meet the requirements of modeling.

#### 3.3.2. Spectral Pretreatment Method Selection

PLSR algorithm was applied to the calibration data set to establish independent models for ferulic acid and Z-ligustilide by relating NIR spectra with reference values analyzed by the HPLC method, and the inﬂuences of the eight pretreatment methods were compared

Eight different pretreatments (MSC, SNV, 1D, 2D, MSC + 1D, MSC + 2D, SNV + 1D, and SNV + 2D) were compared and NIR models based on preprocessed spectra resulted in better prediction than those based on the raw spectra (Table 4).

The best performance of the PLSR model for the quantitation of ferulic acid and Z-ligustilide was obtained based on the preprocessing of 1D. Regarding ferulic acid, an optimal quantification model was developed after the pretreatment of 1D, with latent variables (LV) of 6 and R^2^_C_ and R^2^_P_ of 0.9567 and 0.8580 and RMSEE, RMSECV, and RMSEP of 0.1098, 0.2552, and 0.2658 mg/g, respectively. The PLSR quantitative model of Z-ligustilide was established after the pretreatment of 1D, with LV of 7 and *R*^2^_C_ and *R*^2^_P_ of 0.9611 and 0.8708 and RMSEE, RMSECV, and RMSEP of 0.9611, 2.7046, and 2.7142 mg/g, respectively. Additionally, the RPD values of PLSR models for the determination of ferulic acid and Z-ligustilide were 1.7568 and 1.7392, respectively. The RPD value of the quantitative model constructed after spectral preprocessing was higher than that of the quantitative model based on the original spectrum without preprocessing. Overall, these results showed that the spectral pretreatment method selected in this study improved the performance of the PLSR model for the quantitative analysis of ferulic acid and Z-ligustilide.

#### 3.3.3. Screening of the Spectral Range and Establishment of Optimal PLSR Models

To reduce the redundancy and collinearity caused by unrelated variables and further improve the prediction ability and robustness of the calibration model, Si-PLS was used to screen characteristic spectral interval combinations from the full spectra based on the optimal spectral pretreatment method

The main advantage of this method is that it uses a graphical display to select better subintervals and compare the prediction performance between local models and the full-spectrum model [53]. In this study, the preprocessed full-spectrum region (10,000-4,000 cm^−1^) was first divided into 20 equidistant intervals, and two of each spectral interval were combined to explore their synergistic eﬀects. The segment and largest LV were set to 5 and 15, respectively. All combinations were determined by cross-validation and that with minimum RMSEP was considered the best. Then, the selected important spectral regions were used for recalibration to optimize the performance of initial PLSR models, which were established based on the entire wavenumber span.

**Figure 5:**
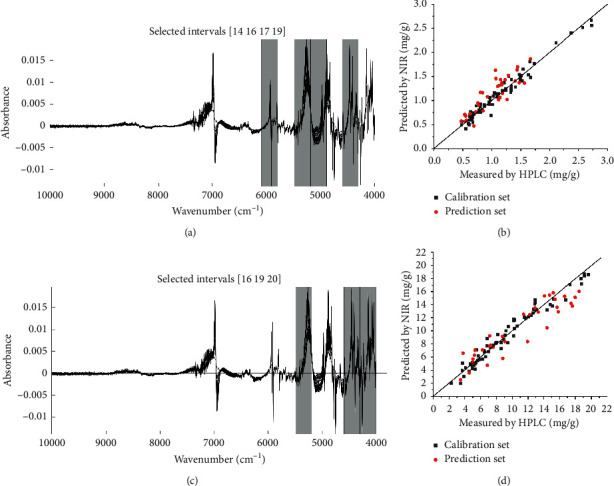
Measured versus predicted plot (b and d) for the concentrations of ferulic acid and Z-ligustilide by the best PLSR models obtained with the optimal spectral interval combinations (a and c). Ferulic acid (a and b), Z-ligustilide (c and d).

The selected characteristic wavenumber ranges and linear regression plots of the measured against predicted values for the best PLSR models developed by selecting spectral intervals in determining the ferulic acid and Z-ligustilide contents are shown in Figure 5 and the parameters of the final optimized PLSR model are summarized in Table 5. For ferulic acid, optimal combinations of spectral intervals selected were [14 16 17 19], which corresponded to 6090.1-5793.11, 5488.42-5191.43, 5187.58-4890.59, and 4589.75-4296.62 cm^−1^, respectively (Figures 5(a) and 5(b)). Regarding Z-ligustilide, optimal combinations of spectral intervals selected were [16 19 20], which corresponded to 5488.42-5191.43, 4589.75-4296.62, and 4292.77-3999.64 cm^−1^, respectively (Figures 5(c) and 5(d)). The common feature of the selected spectral interval combinations for ferulic acid and Z-ligustilide was that they all included the wavenumber ranges of 5488.42-5191.43 cm^−1^and 4589.75-4296.62 cm^−1^. The scatter plot that displays the correlation between the reference measurement and the FT-NIR prediction in the calibration and prediction sets is shown in Figures 5(c) and 5(d). Under the same spectral preprocessing method, the best PLSR models established with selected important spectral regions for the quantitative determination of ferulic acid and Z-ligustilide had higher *R*^2^_P_ (0.9118 and 0.9206, respectively) and RPD (2.1924 and 2.4544, respectively) values and lower RMSEP than those generated with the full spectral range, indicating that these models were more reliable. All the RPD values of the final optimized model that was obtained after preprocessing optimization and spectral range screening were greater than 2, indicating that the two established quantitative models had good prediction stability.

## 4. Conclusion

The current study investigated the feasibility of tracing the geographical origin using FT-NIR coupled with chemometric techniques. The results showed that the SVM classification model of NIR could distinguish the geographical origins of ASR with acceptable precision and accuracy, and a recognition rate of 83.72%. This suggested that the NIR spectroscopy combined with the SVM algorithm could be used as a promising technique to identify Chinese herbal medicines from different places. However, the prediction accuracy was lower than 2.7% of the RF model in the literature [29], implying that the RF is more suitable for the problem of multiclassification than the SVM. Ferulic acid and Z-ligustilide are vital indicators of the quality level of ASR. To further explore the application of NIR in the rapid quality assessment of ASR, the two best PLSR models for fast quantification of ferulic acid and Z-ligustilide were established using optimal spectral pretreatment methods and the most important spectral interval. The final optimal PLSR model exhibited good prediction performance; *R*^2^_P_ for the ferulic acid quantitation model and Z-ligustilide quantitation model was 0.9118 and 0.9206, respectively. The results showed that PLSR is more suitable for the construction of quantitative models than GA-MLR [29]. In brief, the index component coupled with the FT-NIR strategy was a simple and reliable method for rapid quantitative analysis and quality assessment of ASR.

Generally, it could be concluded that, compared with the conventional tedious and time-consuming wet chemistry method, NIR spectroscopy combined with SVM and PLSR algorithms can be exploited in the discrimination of ASR from different geographical locations for quality assurance, quality control, and monitoring. This study may serve as a basis for further geographical trace and quality assessment of ASR and provide a useful way of thinking and offering a reference for quality analysis of agricultural, pharmaceutical, and food products.

## Figures and Tables

**Table 1:** The influence of pretreatment on SVM model.

Pretreatment	C	g	Accuracy of calibration (%)	Accuracy of prediction (%)
RAW	198668	0.00000289	87.69	76.74
MSC	1048580	0.00000166	92.31	81.40
SNV	1048580	0.00000095	90.77	79.07
1D	27.8576	0.00074010	100	79.07
2D	256	0.00001526	89.23	72.09
**MSC** **+** **1D**	**9.18959**	**0.00128858**	**98.46**	**81.40**
MSC + 2D	16.0000	0.00024414	90.77	62.79
SNV + 1D	5.27803	0.00128858	92.31	72.09
SNV + 2D	48.5029	0.00024414	100	81.40

**Table 2:** The results of methodology validation for HPLC analysis.

Analytes	Calibration curves^*a*^	*r* ^2^	Linear range (*μ*g/mL)	Precision (RSD, %, *n* = 6)	Repeatability (RSD, %, *n* = 6)	Stability (RSD, %, *n* = 8)	Recovery (mean, %, *n* = 9)
Ferulic acid	*y* = 51125.819*x* – 11166.578	0.9999	0.5417–54.1728	0.23	1.36	0.58	99.10
Z-ligustilide	*y* = 23458.696*x* – 25427.266	0.9999	3.7418–374.1836	0.23	1.54	0.80	97.00

^a^ *y* is the value of peak area, and *x* is the value of the reference compound's concentration (*μ*g/mL).

**Table 3:** Contents of ferulic acid and Z-ligustilide determined by HPLC.

Analytes	Total set				Calibration set				Prediction set			
	*n*	Range (mg/g)	Mean	SD	*n*	Range (mg/g)	Mean	SD	*n*	Range (mg/g)	Mean	SD
Ferulic acid	99	0.4730–2.7307	1.0900	0.4669	33	0.4790–2.7307	1.1344	0.5243	33	0.4730–1.5626	1.0014	0.3117
Z-ligustilide	99	2.2710–19.6627	9.9453	4.7207	33	2.2710–19.6627	9.5029	4.5500	33	3.4730–18.6352	10.8301	4.9985

**Table 4:** Parameters of PLSR models for the determination of ferulic acid and Z-ligustilide in ASR using FT-NIR based on diﬀerent pretreated methods.

Compounds	Pretreatment	LV^a^.	*R* ^2^_c_	RMSEE	RMSECV	*R* ^2^_p_	RMSEP	RPD
*Ferulic acid*	RAW	6	0.6495	0.3124	0.4736	0.5580	0.3190	1.4639
	MSC	7	0.6591	0.3105	0.3833	0.6080	0.2774	1.6833
	SNV	7	0.6755	0.3029	0.3764	0.6382	0.2549	1.8320
	**1D**	**6**	**0.9567**	**0.1098**	**0.2552**	**0.8580**	**0.2658**	**1.7568**
	2D	4	0.9688	0.0918	0.2611	0.8628	0.2714	1.7204
	MSC + 1D	6	0.9583	0.1078	0.2673	0.8396	0.2935	1.5909
	MSC + 2D	4	0.9645	0.0980	0.2642	0.8416	0.2956	1.5798
	SNV + 1D	6	0.9579	0.1083	0.2669	0.8441	0.2866	1.6292
	SNV + 2D	4	0.9651	0.0971	0.2642	0.8448	0.2918	1.6001

*Z-ligustilide*	RAW	8	0.6167	3.1807	3.9700	0.6261	2.8685	1.6457
	MSC	7	0.6378	3.0677	3.5058	0.6610	2.6056	1.8118
	SNV	7	0.6182	3.1495	3.5172	0.6332	2.7987	1.6868
	**1D**	**7**	**0.9644**	**0.9611**	**2.7046**	**0.8708**	**2.7142**	**1.7392**
	2D	3	0.8678	1.7979	2.8056	0.7428	3.4784	1.3572
	MSC + 1D	7	0.9617	0.9977	2.8443	0.8637	2.7813	1.6973
	MSC + 2D	3	0.8576	1.8660	2.8725	0.7361	3.4780	1.3573
	SNV + 1D	7	0.9616	0.9994	2.8398	0.8634	2.7841	1.6956
	SNV + 2D	3	0.8571	1.8692	2.8687	0.7360	3.4759	1.3581

^a^ LV = latent variable.

**Table 5:** Performance of the optimized PLSR models for the determination of ferulic acid and Z-ligustilide contents in ASR using FT-NIR based on selected spectral intervals combinations and pretreated methods.

Compounds	Pretreatment	Spectral interval	Wavenumber range/cm^−1^	LV	*R* ^2^_c_	RMSEE	RMSECV	*R* ^2^_p_	RMSEP	RPD
Ferulic acid	1D	14, 16, 17, 19	6090.1-5793.11, 5488.42-5191.43, 5187.58-4890.59, 4589.75-4296.62	10	0.9659	0.1052	0.2706	0.9118	0.2130	2.1924
Z-ligustilide	1D	16, 19, 20	5488.42-5191.43, 4589.75-4296.62, 4292.77-3999.64	14	0.9611	1.0069	3.2600	0.9206	1.9233	2.4544

## Data Availability

Majority of the data used in this research are included in the article. Other data can be made available upon request from the first author and corresponding author.

## Supplementary Materials

Supplementary Materials Supplementary Materials. The supplementary data submitted is a graphical abstract. The most suitable pretreatment method was selected from 8 pretreatment methods, and the qualitative and quantitative models of Angelicae Sinensis Radix were constructed by NIR spectroscopy combined with SVM and PLSR algorithm, respectively.
