# Supplementary material for: Rapid Geographical Origin Identification and Quality Assessment of Angelicae Sinensis Radix by FT-NIR Spectroscopy
Source: J Anal Methods Chem. 2021 Jan 12;2021:8875876. doi: 10.1155/2021/8875876 (PMC7815386; doi:10.1155/2021/8875876)
Supplement: Supplementary Materials — The supplementary data submitted is a graphical abstract. The most suitable pretreatment method was selected from 8 pretreatment methods, and the qualitative and quantitative models of Angelicae Sinensis Radix were constructed by NIR spectroscopy combined with SVM and PLSR algorithm, respectively. [file 8875876.f1.docx]

**Supplementary Materials**

# Rapid geographical origin identification and quality assessment of Angelicae Sinensis Radix by FT-NIR spectroscopy

Zhen-yu Zhang,^1^ Ying-jun Wang,^1^ Hui Yan,^*1^ Xiang-wei Chang,^2^ Gui-sheng Zhou,^1^ Lei Zhu,^1^ Pei Liu,^1^ Sheng Guo,^1^ Tina T. X. Dong,^3^ and Jin-ao Duan^*1^

*^1^* *National and Local Collaborative Engineering Center of Chinese Medicinal Resources Industrialization and Formulae Innovative Medicine, and Jiangsu Collaborative Innovation Center of Chinese Medicinal Resources Industrialization,* *Nanjing University of Chinese Medicine, Nanjing 210023, China;*

*^2^ School of pharmacy, Anhui University of Chinese Medicine, Hefei 230012, China;*

*^3^ Division of Life Science and Centre for Chinese Medicine, The Hong Kong University of Science and Technology, Hong Kong, China.*

Correspondence should be addressed to Hui Yan; yanhui@ njucm.edu.cn and Jin-ao Duan; dja@njucm.edu.cn


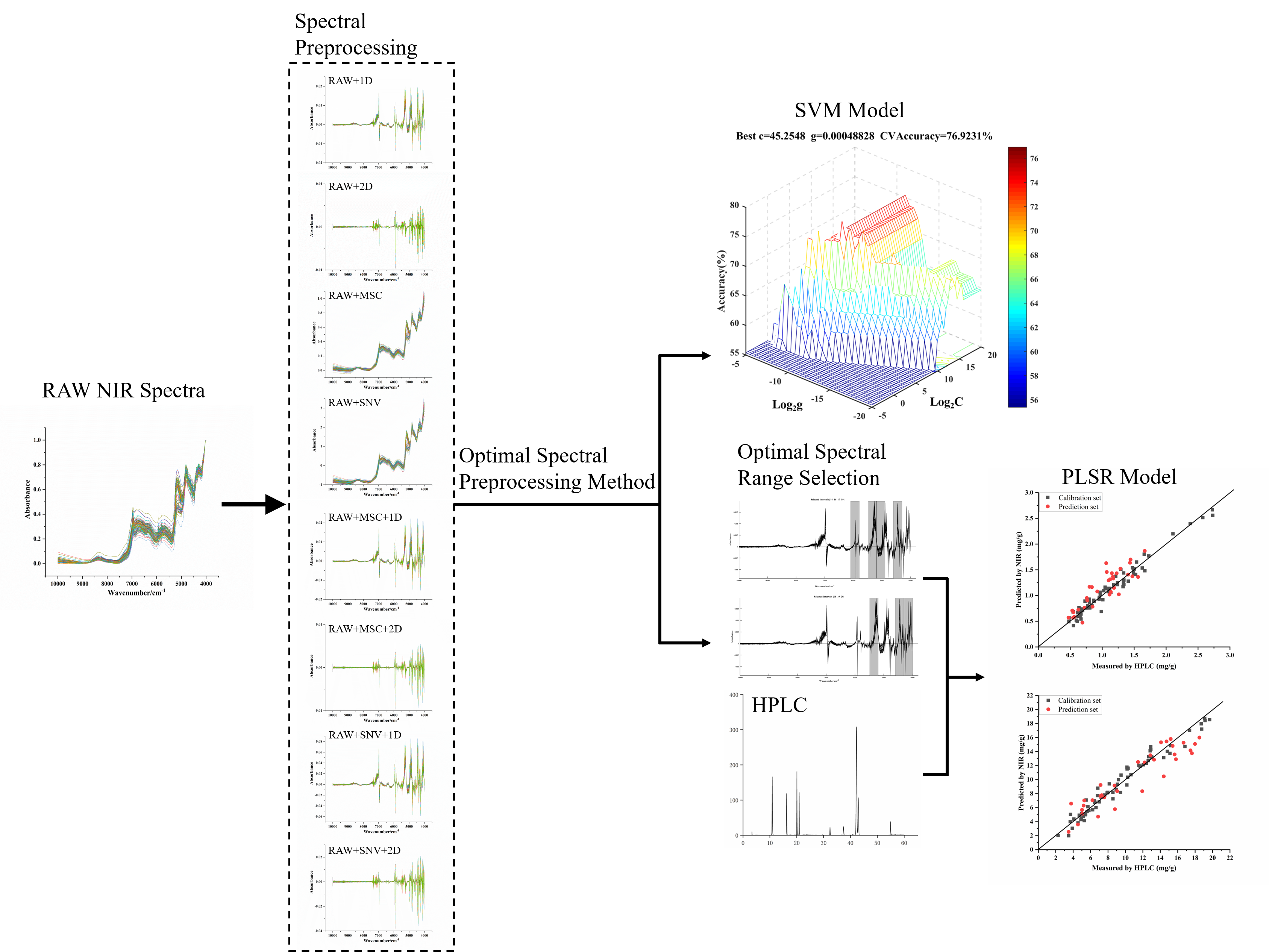


Graphical abstract
